# COVID-19 post-vaccination depression in older Israeli adults: the role of negative world assumptions

**DOI:** 10.1017/gmh.2022.11

**Published:** 2022-01-31

**Authors:** Lee Greenblatt-Kimron, Yaakov Hoffman, Menachem Ben-Ezra, Robin Goodwin, Yuval Palgi

**Affiliations:** 1 School of Social Work, Ariel University, Ariel 40700, Israel; 2 Interdisciplinary Department, Bar-Ilan University, Ramat-Gan, Israel; 3 Department of Psychology, Warwick University, Coventry, UK; 4 Department of Gerontology, University of Haifa, Haifa, Israel

**Keywords:** COVID-19 vaccine, depression, older adults, word assumptions

## Abstract

**Background:**

With the outbreak of severe acute respiratory syndrome coronavirus 2 (SARS-CoV-2) pandemic, the aging population has been shown to be highly vulnerable. As a result, policy makers and the media urged older adults to restrict social interactions, placing them at greater risk of mental health problems, such as depression. However, there has been a little previous attempt to examine coronavirus disease-2019 (COVID-19) vaccine-related risk factors and depressive symptoms amongst older adults.

**Methods:**

Participants (938 older adults, *Mage =* 68.99, s.d. = 3.41, range 65–85) answered an online questionnaire at the start of the COVID-19 vaccination program in Israel. Participants completed measures of background characteristics, world assumptions, COVID-19 vaccine-related variables, and symptoms of depression.

**Results:**

Univariate logistic regression revealed that more negative world assumptions were linked with clinical depression levels.

**Conclusions:**

Older adults in our sample were susceptible to unique factors associated with clinical depression influenced by their world assumptions during their COVID-19 vaccination. The high level of depression following vaccination indicates that it may take time to recover from depression associated with pandemic distress. Cognitive interventions that focus on world assumptions are recommended.

## Introduction

With the outbreak of the severe acute respiratory syndrome coronavirus 2 (SARS-CoV-2) pandemic, it was postulated that the pandemic be considered a (traumatic) stressor (Shevlin *et al*., 2020). Old age was confirmed as a risk for coronavirus disease-2019 (COVID-19) complications (Palgi *et al*., 2020); therefore, policy makers and media urged older adults to restrict social interactions (Ayalon, 2020). Consequently, evidence from France, Italy, and Spain, the first countries outside Asia affected by the virus, revealed increased depressive feelings during the lockdown in the older population in April 2020, approximately 1 month after the beginning of the first lockdown (Arpino *et al*., 2021). Likewise, older adults in Israel reported peritraumatic distress (Greenblatt-Kimron *et al*., 2021) and depressive symptoms during COVID-19 (Palgi *et al*., 2020) in the initial stage of the lockdown in Israel between March 15^th^ and April 1^st^ 2020. Despite vaccination programs (potentially) signaling the ‘light at the end of the pandemic tunnel' (Hoffman *et al*., 2021*a*), research reported strong links tying vaccination side effects with depression and high prevalence of vaccination hesitancy with anxiety, depression, peritraumatic distress (Palgi, *et al*., 2021*a*) and post-traumatic stress disorder even in those who were vaccinated (Palgi, *et al*., 2021*b*), suggesting that many older adults continue to be psychologically impacted. However, the association between psychological health and postvaccination depression in older adults has been little explored.

Depression is a prevalent mental disorder among older adults, which commonly causes substantial distress and reduced quality of life (Kok and Reynolds, 2017; Overvliet *et al*., 2021). The association found between the pandemic and depression in older adults (Palgi *et al*., 2020; Arpino *et al*., 2021) can result from appraisals of traumatic events crucial to emotional responses and consequent psychological reaction (Lazarus, 1993). Cognitive processes have been found to maintain post-traumatic stress symptoms and depression in old age (Greenblatt-Kimron and Cohen, 2020). Beck's (2008) cognitive Developmental Model of Depression suggests that depressive symptoms manifest from negative interpretations of life events, which are rooted in maladjusted beliefs concerning the self, others and the world developed from early life experiences. In line with Beck's (2008) model, a study among older care givers found negative thought patterns to be associated with postvaccination depression (Segerstrom *et al*., 2008). One possible direction is that one may have adopted negative world assumptions (Janoff-Bulman, 1992), which are cognitive schemas that create a person's assumptive world regarding the self, others and the world. According to Janoff-Bulman (1992) these assumptions are inherently optimistic; nevertheless, they may be shattered by stressful life events, in this case, the COVID-19 global pandemic. People may then attempt to rebuild their assumptive worlds by incorporating the stressful experience (Janoff-Bulman, 1992), as demonstrated in a recent study among older adults (Greenblatt-Kimron, 2021). Research in Poland on the COVID-19 pandemic found an association between a positive world view and fewer panic thoughts and emotions evoked by the ongoing pandemic (Trzebiński *et al*., 2020). Moreover, older adults with internalized negative attitudes such as an older subjective age identity showed more adverse effects of loneliness during the COVID-19 pandemic (Shrira *et al*., 2020).

The rapid production of COVID vaccines may have likely caused mistrust about the safety, credibility and effectiveness of the vaccines that was enhanced by anti-vaccination movements (Shacham *et al*., 2021). Indeed, preliminary results in Israel indicate that vaccine hesitancy was widespread in Israel with negative attitudes toward the COVID-19 more prevalent than attitudes toward general vaccines (Shacham *et al*., 2021). Therefore, it is assumed that alongside the hope that the vaccination project brought with it, many negative psychological reactions may have been elicited by the vaccination given to participants in this study (i.e. Pfizer-BioNTech), as such vaccinations adopted a new technology that was not used in the past.

Based on the above, and in conjunction with a preliminary study reporting high depression levels among Israelis vaccinated for COVID-19 (Palgi *et al*., 2021*a*), the current study aimed at understanding the cognitive processes associated with clinical depression among vaccinated older adults, with a particular focus on negative schemas, specifically negative world views. We predict that negative world assumptions will be associated with depressive symptomology in vaccinated older adults.

## Methods

### Participants and procedure

Data were collected among older Jewish adults across Israel after Israel initiated its COVID-19 vaccine program. We employed a survey company using a web-based platform with representative proportional sampling, with data collected between January 25 and 4 February 2021. On the final day of data collection, Israel had completed more than 5.1 million vaccine doses, out of which about 1.9 million Israeli's had received the second dose, and 84% of those aged 60+ had received at least one of the two Pfizer doses (Israeli Ministry of Health). After omitting 42 participants who were not vaccinated, and 38 participants who did not report depressive symptom levels, the remaining 938 older adults had all received at least one dose of the COVID-19 vaccine (mean age = 68.99, s.d. = 3.41, range 65–85). Most participants were women (*n* = 562, 59.9%), married/with a partner (*n* = 709, 75.6%), and had tertiary education and above (*n* = 725, 77.3%). All participants provided informed consent. Ethical approval was granted by the Ariel University Institutional Review Board (ref no. AU-SOC-MBE-20191029-2).

### Measures

*Background Characteristics* included age, sex, marital status (1 = not married, 2 = married or living in cohabitant), level of religiosity, and education (two levels 1 = up to secondary education, 2 = tertiary education and above).

*World assumptions* were measured by four items developed for this study, based on the items suggested by (Blevins *et al*., 2016). These items evaluated effects of shattered assumptions, which according to Janoff-Bulman (1992) may be due to stressful life events (i.e. the global pandemic); these world assumptions items state: *(I feel that my life has no meaning or purpose, I fear a negative event that may occur in the future, I feel like I have less control over my life, the world is a dangerous place to live in).* Participants rated their basic assumptions regarding the world, other people and self since the initiation of the COVID-19 vaccine program in Israel. Responses were rated on a five-point Likert scale ranging from 1 (*not at all*) to 5 (*to a very great degree*). A higher score indicates a more negative world assumption. Cronbach's *α* in the current study was 0.810.

*Vaccination measures* included the number of days since the first vaccine and vaccine hesitancy (8 adapted items taken from Giambi *et al*., 2018). Cronbach's *α* in the current study was 0.832.

*Depressive symptoms* were measured with a 9-items scale (PHQ-9, Kroenke and Spitzer, 2002), which indicates clinically significant depression with good reliability and construct validity (Kroenke and Spitzer, 2002). Participants rated how often they had been bothered by any of the given problems since the initiation of the COVID-19 vaccine program on a four-point Likert scale from 1 (*not at all*) to 4 (*nearly every day*). Scores range from 0 to 28 and are categorized from no depression to mild (0–9) or moderate-to-severe depression (10 and above). Cronbach's *α* in the current study was 0.846

### Data Analysis

A univariate logistic regression analysis was conducted using the IBM statistic package (SPSS-25) software to predict depression (See Table 1). We controlled for age, sex, marital status, level of religiosity, and level of education, subjective health, COVID-19 related exposure, and added vaccine hesitancy, days since vaccination and world assumptions to the equation. Odds ratio (OR) with 95% confidence interval (CI) indicated the independent association of each correlate with each outcome.

**Table 1.** Logistic regression analyses predicting the likelihood of clinical depression

	B	s.e.	Odds ratio	95.0% CI for Odds ratio	
				Lower	Upper
Age	0.01	0.04	1.01	0.93	1.09
Sex^a^	1.30	0.32	3.67***	1.97	6.82
Marital status^b^	−0.19	0.19	0.83	0.58	1.20
Education^c^	−0.62	0.30	0.94	0.52	1.70
Religiosity	0.06	0.29	1.06	0.60	1.87
Subjective health	−0.55	0.14	0.58***	0.44	0.76
COVID-19 related exposure	0.19	0.25	1.21	0.74	1.98
Vaccine hesitancy	0.17	0.16	1.18	0.86	1.63
Days since vaccine	−0.00	0.01	1.00	0.97	1.03
World assumptions	1.38	0.15	3.98***	3.00	5.29
Constant	−6.20	2.99	0.00*		

*Note:* Total *N* *=* 938; Nagelkerke *R*^2^ = 0.35, clinical depression = 103, negative depression = 831.

a Sex 1 = male, 2 = female.

b Marital status 1 = not married, 2 = currently married, or living with a partner.

c = two education levels 1 = up to secondary education, 2 = tertiary education and above.

* *p* < 0.05 ***p < 0.001.

## Results

As Shown in Table 1, 103 (11.0%) of the participants reached the criteria for clinical depression after being vaccinated with at least one dose of a COVID-19 vaccine. Logistic regression analysis was employed to predict the probability that older adults would reach clinical depression levels. The full model containing all predictors was statistically significant (*χ*^2^(10, *N* = 935) = 197.20, *p* < 0.001). The model as a whole explained between 19% (Cox and Snell R square) and 38.0% (Nagelkerke R squared) of the variance in clinical depression.

A univariate logistic regression analysis indicated that females had a 3.6 times higher chance of suffering from clinical depression than males after receiving one dose of the COVID-19 vaccine. Better subjective health was associated with a 50% lower chance of experiencing depressive symptoms. Findings for all other covariates were non-significant (all *p* > 0.05). Each score on the independent variable of more negative world assumptions rendered one 4 times more likely to have clinical depression (OR 3.98, 95%, CI 2.99–5.28). For further information see Table 1.

## Discussion

The current study showed an association between negative world assumptions and clinically relevant depressive symptoms amongst older vaccinated adults. To the best of our knowledge, this study is the first study to find such as association amongst older vaccinated adults during the coronavirus vaccine rollout during the pandemic.

Those who reported negative world assumptions reported 4.4 times higher odds for clinical symptoms of depression than those with more positive assumptions. Beck's Developmental Model of Depression (Beck, 2008) claims that depressive symptoms emerge from negative interpretations of life events (i.e. the COVID-19 pandemic). These are rooted in maladjusted beliefs concerning the self, others and the world, developed from early life experiences and may have persisted latently until being triggered by the coronavirus pandemic. These are then manifested in negative thoughts that augment depressive symptoms even after receiving the COVID-19 vaccination. Older adults may have also internalized the widespread negative attitudes toward older people during the COVID-19 pandemic (see Ayalon, 2020), which may have affected their world assumptions, or triggered latent negative cognitions. The COVID-19 pandemic has been considered a robust stressor (Shevlin *et al*., 2020). Even after receiving a vaccination that is supposed to restore the world to being the ‘safe’ place, older adults who hold negative world assumptions may be unable to perceive the world as secure and feel protected from the virus; and therefore, experienced depression (11.0%). Such older adults may have adopted a negative world view of ‘what-was-will-be’ (see Hoffman *et al*., 2021*b*) common after widespread stressors. In line with the suggestion of Hoffman *et al*. (2021*b*), this may express a view that equilibrium may take time to reconstruct, despite the initiation of vaccine programs.

We also note that health awareness increased during the COVID-19 pandemic (Shacham *et al*., 2021). Attitudes toward the COVID-19 vaccination were more negative compared to attitudes regarding other vaccines (Shacham *et al*., 2021). It may thus be that more negative world assumptions may have resulted in a lack of faith in the vaccine. The rollout of the Pfizer COVID-19 vaccine was in its nascent stage during our data collection, with conspiracies and mistrust ripe about the short amount of time for vaccination development and long-term safety, credibility and effectiveness of COVID-19 vaccines (Shacham *et al*., 2021). While the vaccine should have augured hope, negative cognitions may have elicited feelings of helplessness and more wariness of vaccinations and; therefore, these negative cognitions were linked with depression.

Despite the above, our current study had several limitations. First, we used a cross-sectional design making it difficult to determine causality. Therefore, the receipt of the vaccine may only be significant at this particular timepoint (influencing negative world views). Longitudinal studies that examine the associations between pre-existing world assumptions and depression before and after vaccination are recommended. We used a self-report design and lacked a psychiatric evaluation of participants. Finally, we used a web-based sample, which may be biased toward older adults with digital literacy (Nimrod, 2018), and may also underrepresent minor, but sizable, groups of Israeli older adults (e.g. Israeli Arabs, immigrants from the former Soviet Union).

Nevertheless, the associations found in the current study between negative world assumptions with depressive symptoms among older adults have important implications for practitioners during the global implementation of a COVID-19 vaccine. On a practical level, the knowledge gained from this study may be utilized to build appropriate treatment interventions based on a cognitive approach directed at processing clients' world assumptions, and in this way increase a sense of belief in self, in others and in the world. More positive world assumptions could decrease depressive symptomology in older adults during global pandemic vaccination programs, with these are critical for global success in combating the virus.
